# Deletion of TNF in Winnie-*APC^Min/+^* Mice Reveals Its Dual Role in the Onset and Progression of Colitis-Associated Colorectal Cancer

**DOI:** 10.3390/ijms232315145

**Published:** 2022-12-02

**Authors:** Giulio Verna, Marina Liso, Elisabetta Cavalcanti, Raffaele Armentano, Alessandro Miraglia, Vladia Monsurrò, Marcello Chieppa, Stefania De Santis

**Affiliations:** 1 Department of Pharmacy, University of Salerno, 84084 Fisciano, Italy; gxv71@case.edu; 2 Digestive Health Research Institute, Case Western Reserve University School of Medicine, Cleveland, OH 44106, USA; 3 National Institute of Gastroenterology “S. de Bellis”, IRCCS Research Hospital, 70013 Castellana Grotte, Italy; marina.liso@irccsdebellis.it (M.L.); elisabetta.cavalcanti@irccsdebellis.it (E.C.); raffaele.armentano@irccsdebellis.it (R.A.); 4 Department of Biological and Environmental Sciences and Technologies, University of Salento, 73100 Lecce, Italy; alessandro.miraglia@unisalento.it (A.M.); marcello.chieppa@unisalento.it (M.C.); 5 Department of Medicine, University of Verona, 37134 Verona, Italy; vladia.monsurro@univr.it; 6 Department of Pharmacy-Pharmaceutical Science, University of Bari Aldo Moro, 70125 Bari, Italy

**Keywords:** colon cancer, inflammation, animal models, ulcerative colitis, TNF

## Abstract

Colorectal cancer (CRC) is among the best examples for depicting the relationship between inflammation and cancer. The introduction of new therapeutics targeting inflammatory mediators showed a marked decrease in the overall risk of CRC, although their chemopreventive potential is still debated. Specifically, a monoclonal antibody that blocks tumor necrosis factor (TNF), infliximab, increases CRC risk in inflammatory bowel disease patients. To address the axis between TNF and CRC development and progression, we depleted the *Tnf* from our previously established murine model of colitis-associated cancer (CAC), the Winnie-*Apc^Min/+^* line. We characterized the new Winnie-*APC^Min/+-^*TNF-KO line through macroscopical and microscopical analyses. Surprisingly, the latter demonstrated that the deletion of *Tnf* in Winnie-*Apc^Min/+^* mice resulted in an initial reduction in dysplastic lesion incidence in 5-week-old mice followed by a faster disease progression at 8 weeks. Histological data were confirmed by the molecular profiling obtained from both the real-time PCR analysis of the whole tissue and the RNA sequencing of the macrodissected tumoral lesions from Winnie-*APC^Min/+-^*TNF-KO distal colon at 8 weeks. Our results highlight that TNF could exert a dual role in CAC, supporting the promotion of neoplastic lesions onset in the early stage of the disease while inducing their reduction during disease progression.

## 1. Introduction

In recent years, the incidence and mortality of cancer have been rapidly increasing worldwide, particularly in industrialized countries [1]. Several environmental factors, including pollution, smoking, diet, breastfeeding, antibiotics, infections/vaccinations, and childhood hygiene, may play a role and can partially explain this observation, but the concurrence of inflammation and cancer cannot be ignored as these two pathogenic processes appear to be linked [2,3]. In support of this concept, the introduction of non-steroidal anti-inflammatory drugs for the treatment of chronic inflammatory disorders has been shown to protect patients from an increased risk of developing various forms of cancer [4]. Moreover, epidemiological studies have also demonstrated that chronic inflammation predisposes affected individuals to different types of cancer, resulting in the inclusion of inflammation as the seventh hallmark feature of cancer [5]. In detail, before the introduction of effective therapeutic options for controlling chronic inflammation in the gastrointestinal tract, inflammatory bowel disease (IBD) has been associated with an increased risk of developing colorectal cancer (CRC) [6]. Specifically, multiple epidemiological studies have described an increased risk of CRC in the first decade after an ulcerative colitis (UC) diagnosis [7]; this is especially true for patients diagnosed with UC during childhood, adolescence, or young adulthood, particularly for male patients [8,9].

Interestingly, the introduction of new therapeutic options that target either immune responses or inflammatory mediators has been shown to markedly decrease the overall risk of CRC [10,11], even though the chemopreventive potential of 5-aminosalicylic acid (5-ASA) and thiopurines is still under debate [12,13]. Recently, biological agents have been introduced for the induction as well as maintenance of disease remission in IBD patients [14]. In Europe, one of the approved and most clinically-utilized molecules of these biologics is infliximab (IFX), a monoclonal antibody that specifically blocks tumor necrosis factor (TNF) [15]. In addition to IFX, several other biologics targeting inflammatory cytokines have been investigated in recent years as a second-line therapy for IFX-unresponsive patients [16,17,18,19,20].

However, conflicting reports exist concerning the relationship between IFX-based therapy and an increased risk of CRC and other forms of cancer in IBD patients receiving such therapy [21,22]. The dual nature of TNF’s bioactivity, which is responsible for its paradoxical anti- and pro-tumorigenic effects, depends on the cell type expression and response, environment, dose, and other factors [23]. On the one hand, TNF exerts its anti-tumorigenic potential through its interaction with the death domain-containing proteins and caspases, inducing apoptosis [24]. On the other hand, TNF possesses pro-tumorigenic activity as a result of the nuclear factor kappa-light-chain-enhancer of activated B cells (NF-κB) activation and mitogen-activated protein kinase (MAPK) pathways that are associated with both inflammation and carcinogenesis [25,26,27]. To dissect the role of TNF in colitis-associated cancer (CAC) development and progression, we took advantage of our previously established mouse model, the Winnie-*Apc^Min/+^* model [28,29], and combined an inflammatory background with a genetic predisposition to small intestinal polyposis.

In the present study, we addressed the impact of TNF on the development and progression of CAC by genetically depleting *Tnf* from Winnie-*Apc^Min/+^* mice and characterizing the development of neoplastic lesions from the histological and molecular points of view in the new Winnie-*Apc^Min/+^*-TNF-KO murine line. Our results demonstrate a dual role for TNF in CAC pathogenesis thanks to its ability to support the promotion of neoplastic lesions onset at the early stage of the disease and its ability to reduce their incidence during disease progression.

## 2. Results

### 2.1. The Absence of TNF Does Not Protect 5-Wk-Old Winnie-Apc^Min/+^-TNF-KO Mice from the Development of Dysplastic Lesions

**Figure 1:**
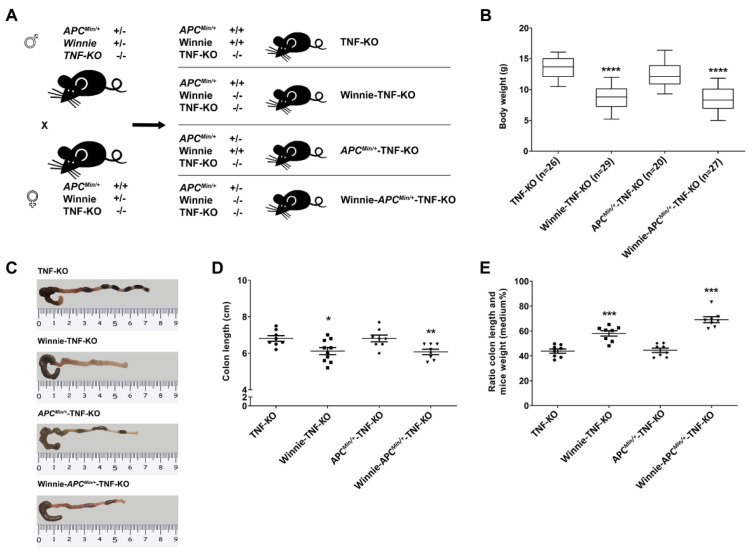
Creation and characterization of the Winnie-*APC^Min/+^*-TNF-KO murine line. (**A**) Breeding strategy used to obtain Winnie-*APC^Min/+^*-TNF-KO mice along with the control littermates. (**B**) Body weight analysis of 4-wk-old mice from different genotypes. Representative images of colons (**C**), data analysis of colon length (**D**), and colon length adjusted to the body weight (**E**) for Winnie-*APC^Min/+^*-TNF-KO mice and control littermates. D-E: eight animals/group. Box plots represent the mean ± SEM. * *p* < 0.05, ** *p* < 0.01, *** *p* < 0.001, **** *p* < 0.0001 compared with TNF-KO mice. TNF: tumor necrosis factor.

Based on the importance of TNF in IBD pathogenesis and on the established efficacy of anti-TNF biologics in clinical practice, and taking advantage of our previous established model of CAC (the Winnie-*Apc^Min/+^* model [28]), we developed Winnie-*Apc^Min/+^* mice lacking TNF by crossing commercially-available *Tnf* null mice (line TNF-KO) [30] with Winnie-*Apc^Min/+^* mice. The intent was to create the Winnie-*Apc^Min/+^*TNF-KO model, and we used the same breeding strategy that was used to generate Winnie-*Apc^Min/+^* mice, i.e., double heterozygote mutations for males (on Apc and Muc2 genes for *Apc^Min/+^* and Winnie strains, respectively) and a single heterozygote mutation (Muc2) for females. In addition, a *Tnf* null mutation in a homozygous state was present on both breeders to generate Winnie-*Apc^Min/+^*-TNF-KO mice and control littermates (TNF-KO, Winnie-TNF-KO, and *Apc^Min/+^*-TNF-KO strains; Figure 1A).

As previously described for Winnie-*Apc^Min/+^* mice [31], the deletion of TNF did not protect Winnie-*Apc^Min/+^*-TNF-KO mice from the reduction in body weight, colon length, and their relative ratio resulting from the missense mutation in Winnie mice (Figure 1B–E). Dysplastic aberrant crypt foci (ACFs) were also detected along the colon length of 5-wk-old Winnie-*Apc^Min/+^*-TNF-KO mice, with an increased frequency moving distally towards the rectum (Table 1 and Supplementary Figure S1), even if their incidence, multiplicity, and tumor grading were substantially reduced compared with age-matched Winnie-*Apc^Min/+^* mice [28] (Table 1).

Moreover, the absence of TNF did not protect Winnie-*Apc^Min/+^*-TNF-KO from the previously described inflammatory signs of Winnie-*Apc^Min/+^* mice. Specifically, epithelial erosion (Supplementary Figure S1) and a decrease in mucin expression in dysplastic areas were also present in Winnie-*Apc^Min/+^*-TNF-KO mice and the parental line Winnie-TNF-KO (Supplementary Figure S2). These data demonstrated that, despite the importance of TNF in the intestinal inflammatory environment and consequently in CAC development, the genetic mutation that reduced TNF production did not impair dysplastic lesion development. 

### 2.2. Deletion of TNF Increases Multiplicity and Tumor Grading of Dysplastic ACFs in 8-wk-Old Winnie-Apc^Min/+^-TNF-KO Mice

**Figure 2:**
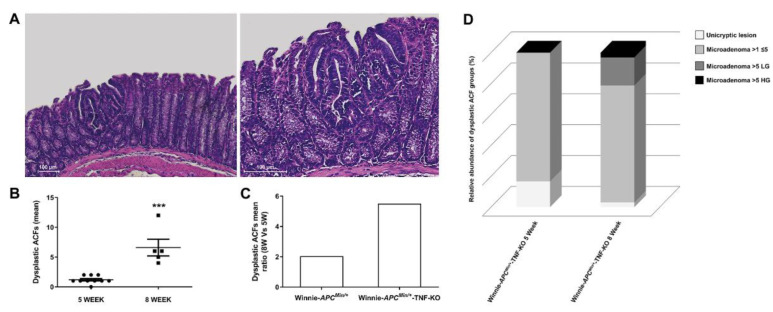
Histological analysis of Winnie-*APC^Min/+^*-TNF-KO mice at 5 and 8 weeks. (**A**) Hematoxylin and eosin staining on 3 μm distal colon sections of 8-wk-old Winnie-*APC^Min/+^*-TNF-KO mice. Images were captured at 10X (left) and 20X magnifications (right). CAC progression from 5 to 8 weeks was calculated for Winnie-*APC^Min/+^*-TNF-KO mice as the mean of dysplastic ACFs (**B**) and reported as the mean ratio between 8 and 5 weeks for Winnie-*APC^Min/+^*-TNF-KO mice and the already published Winnie-*APC^Min/+^* model [28] (**C**). Data reported in B as dot plots represent the mean ± SEM. (**D**) Histograms reporting dysplastic ACFs composition in terms of dimension and grading for Winnie-*APC^Min/+^*-TNF-KO mice at 5 and 8 weeks; 5 weeks: n = 9–10 animals/group; 8 weeks: n = 5–8 animals/group. *** *p* < 0.001. TNF: tumor necrosis factor; CAC: colitis-associated cancer; ACFs: aberrant crypt foci.

In line with the multi-step nature of CRC, we histologically characterized Winnie-*Apc^Min/+^*-TNF-KO mice at a later time point, confirming the expected progression in the number of ACFs at 8 weeks (Figure 2A,B). 

Surprisingly, the presence of ACFs in Winnie-*Apc^Min/+^*-TNF-KO mice was remarkably increased from 5 to 8 weeks of age, with a rate of progression greater (mean ratio 8W vs. 5W: 5.5, Figure 2C) than the published Winnie-*Apc^Min/+^* model (mean ratio 8W vs. 5W: 2.2 [28]). Together with the increased rate of progression, the type of ACFs and their tumor grades also altered over time. Specifically, small neoplastic lesions (unicryptic lesions and microadenoma >1≤5 crypts LG) less frequently appeared at a later time point, whereas microadenoma >5 crypts LG and microadenoma >5 crypts HG were observed instead when comparing 5-week-old to 8-week-old Winnie-*Apc^Min/+^*-TNF-KO mice (Supplementary Table S1 and Figure 2D). This progression, already identified in Winnie-*Apc^Min/+^* mice [28], was more dramatic in Winnie-*Apc^Min/+^*-TNF-KO mice, in which microadenoma >5 crypts LG and HG lesions became evident only at 8 weeks (Table 1, Supplementary Table S1 and Figure 2D). Thus, unexpectedly, the histological analysis of the Winnie-*Apc^Min/+^*-TNF-KO colon at 8 weeks showed a marked increase in the multiplicity, dimension, and grading of dysplastic ACFs, even more than what was observed in Winnie-*Apc^Min/+^* mice [28] (Figure 2 and Supplementary Table S1). 

### 2.3. Molecular Profiling of Whole Tissue and Macrodissected Tumoral Lesions Reveals the Activation of Cancer-Related Pathways with a More Aggressive Phenotype in 8-Wk-Old Winnie-Apc^Min/+^-TNF-KO Mice

**Figure 3:**
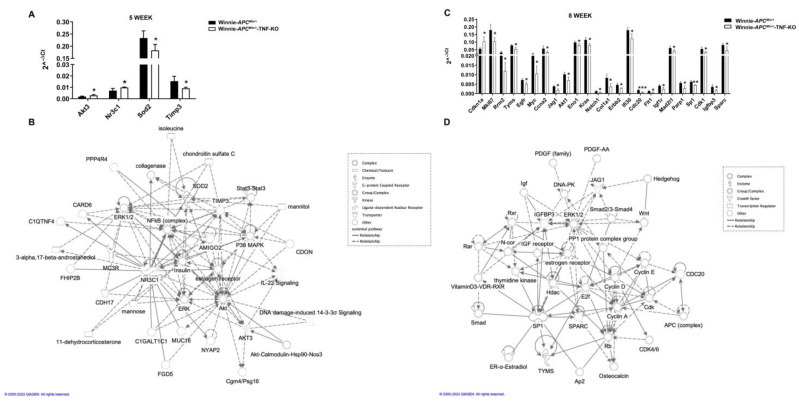
TNF-independent molecular profiling of distal colon from Winnie-*APC^Min/+^* mice during CAC progression. Real-time quantification of Winnie-*APC^Min/+^* (black bars) and Winnie-*APC^Min/+^*-TNF-KO mice (white bars) mRNA expression at 5 weeks (**A**) and 8 weeks (**B**) using pre-custom plates for colorectal cancer (n = 4 animals/group). Histograms represent the mean ± SEM. * *p* < 0.05, ** *p* < 0.01, *** *p* < 0.001. Network analysis of the differential expressed genes at 5 weeks (**C**) and 8 weeks (**D**). The network was algorithmically constructed by IPA software based on the functional and biological connectivity. TNF: tumor necrosis factor; CAC: colitis-associated cancer; IPA: ingenuity pathway analysis.

To dissect the role of TNF ablation during CAC progression at the molecular level, we analyzed the expression of 89 selected genes for CAC in the distal colons of 5- and 8-wk-old Winnie-*Apc^Min/+^*-TNF-KO mice as compared with Winnie-*Apc^Min/+^* mice (Figure 3).

At 5 weeks, we identified four differentially expressed genes (two up- and two down-modulated genes: *Akt3*-*Nr3c1* and *Sod2*-*Timp3*, respectively), while at 8 weeks, the number of genes dramatically increased up to twenty-four (one up- and twenty-three down-modulated genes) (Figure 3A,B). Surprisingly, 23 out of 24 genes coding for oncogenes and tumor-promoting genes were down-modulated; the single up-modulated gene was the cyclin-dependent kinase inhibitor 1A gene (*Cdkn1a*), a target of the tumor suppressor p53. Network analyses of the differentially expressed genes revealed the role of numerous interconnected genes in the free radical scavenging pathway and metabolic disease at 5 weeks and in cancer and cell death/survival pathways along with organismal injury at 8 weeks (Figure 3C,D, respectively). Importantly, the pathway analysis revealed that the absence of TNF at early time points resulted in the induction of alternative inflammatory pathways, while the genes that significantly modulated at 8 weeks were primarily involved in cancer-related pathways (Figures S3 and S4, respectively). 

**Figure 4:**
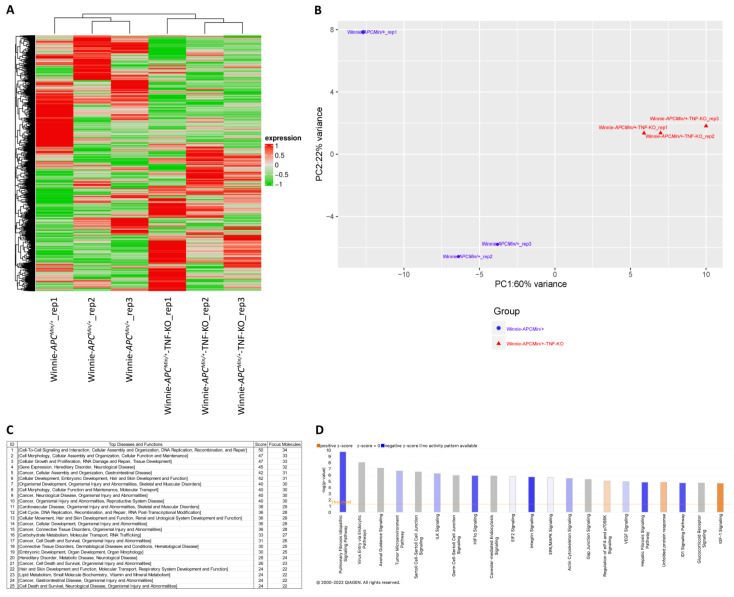
RNA sequencing analysis of macrodissected ACFs from Winnie-*Apc^Min/+^*-TNF-KO and Winnie-*Apc^Min/+^* distal colons. Two-dimensional hierarchical clustering (**A**) and PCA (**B**) analyses based on the significantly differentially modulated genes (FDR<0.05 and fold change threshold ≥1.5 and ≤−1.5) discriminating Winnie-*Apc^Min/+^*-TNF-KO and Winnie-*Apc^Min/+^* ACFs. Top 25 diseases and functions (**C**) and 21 top canonical pathways (**D**) of the genes differentially expressed between the two groups were generated by the IPA. Where an activity pathway was available and a prediction z-score was associated to the canonical pathway, a color code was added to the bar graph (blue: positive; orange: negative). TNF: tumor necrosis factor; PCA: principal component analysis; IPA: ingenuity pathway analysis.

Starting from the data obtained from the whole tissue, we decided to focus on the molecular profiling of the tumoral lesions. Thus, we performed a sequencing analysis on ACFs macrodissected from 8-wk-old Winnie-*Apc^Min/+^*-TNF-KO mice and compared these data with those obtained from the sequencing of Winnie-*Apc^Min/+^* mice of the same age. An unsupervised hierarchical clustering of the gene expression data showed distinct segregation between RNA from Winnie-*Apc^Min/+^*-TNF-KO and Winnie-*Apc^Min/+^* ACFs (Figure 4A). 

In accordance with the results of the unsupervised hierarchical clustering, a clear segregation was noted by the principal component analysis (PCA, Figure 4B). A total of 1266 genes resulted to be differently expressed between the two datasets (FDR < 0.05 and fold change threshold ≥1.5 and ≤−1.5). The differentially expressed genes between Winnie-*Apc^Min/+^*-TNF-KO and Winnie-*Apc^Min/+^* mice were analyzed by the ingenuity pathway analysis (IPA). The IPA core analysis revealed that the top diseases and functions associated with the dataset were deeply related to cancer and its features, cellular assembly and organization, and gastrointestinal disease (Figure 4C). Similarly, the top 25 canonical pathways were linked to carcinogenesis, tumor microenvironment, and cellular remodelling (Figure 4D). These findings supported the hypothesis of having a more aggressive phenotype for the tumoral lesions in Winnie-*Apc^Min/+^*-TNF-KO mice, in accordance with the presence of the “advanced malignant tumor” category in disease and functions being among the top regulator effect networks identified by the IPA (Supplementary Table S2). Moreover, the IPA analysis identified TGFβ among the possible upstream regulators capable of explaining the profile resulting from the comparison between the Winnie-*Apc^Min/+^*-TNF-KO and Winnie-*Apc^Min/+^* mice (Table 2).

In line with the IPA prediction of inhibition for this gene, TGFβR2 resulted to be highly down-modulated in Winnie-*Apc^Min/+^*-TNF-KO as compared with Winnie-*Apc^Min/+^* mice (fold change: −2.01; padj: 7.18 × 10^−6^).

## 3. Discussion

Colorectal cancer is among the best examples for depicting the relationship between inflammation and increased cancer risk. Despite the use of therapies for better control of the inflammatory *status* and the use of routinary endoscopic screening in adult IBD patients for efficiently reducing CRC risks, epidemiological studies still reveal the association between IBD and CRC [32,33,34]. Of note, the axis between intestinal inflammation and cancer development is especially valid for patients diagnosed with IBD during pediatric years, particularly for UC patients [7,8,35].

In the present study, we analyzed the role of TNF in CAC onset and progression by taking advantage of our previous murine model of UC-induced CAC, the Winnie-*Apc^Min/+^* line, which is able to recapitulate the pathology and clinical progression in humans. The Winnie-*Apc^Min/+^* model demonstrates that mild but chronic intestinal inflammation is sufficient to support tumor development in genetically susceptible subjects, giving the advantage of fully dissecting the cascade of events leading to CAC onset and progression. Of note, our previously established model could also be useful for designing alternative therapies by testing new predictive biomarkers for early CAC diagnosis in IBD patients. The lack of response to biological agents (e.g., infliximab) is one of the major drawbacks of this new frontier of drugs with an enormous financial cost for the health system; this is especially true for young UC patients that escape the endoscopic screening due to the presence of flat, rather than polypoid, adenomas. To resemble this subgroup of patients, we created the new Winnie-*Apc^Min/+^*-TNF-KO murine line by depleting the expression of *Tnf* from the Winnie-*Apc^Min/+^* line. 

Our results demonstrated that the deletion of TNF did not ameliorate the phenotype induced by the Winnie mutation but rather increased the frequency of dysplastic lesions in the distal tract of the large intestine relative to the control littermates. Comparing the tumorigenic potential of Winnie-*Apc^Min/+^*-TNF-KO mice with the already published Winnie-*Apc^Min/+^* model, a different modulation for the incidence, multiplicity, and tumor grading of the tumoral lesions was reported. Surprisingly, we demonstrated that the absence of TNF protected Winnie-*Apc^Min/+^*-TNF-KO mice from the early onset of dysplastic lesions at 5 weeks (we detected only small lesions with a low multiplicity and moderate grade of dysplasia), whereas it induced an exacerbated progression of dysplastic lesions during the transition from early to intermediate time points (8 weeks). These results were supported by the molecular analysis of the whole colon from Winnie-*Apc^Min/+^*-TNF-KO mice relative to the Winnie-*Apc^Min/+^* mice, which showed the modulation of the free radical scavenging and metabolic disease signaling at early time points. In line with the literature, both these diseases and functions support an early onset of CRC [36,37]. Furthermore, a marked shift toward the tumorigenic signaling at intermediate time points was reported. Among the higher number of genes that resulted modulated at 8 weeks as compared with the 5 weeks, all were down-modulated except for *Cdkn1a*, which showed an up-modulation. It is possible to speculate that p21 encoded by *Cdkn1a* could either act as an oncogene or tumor suppressor gene depending on the cellular context, subcellular localization, and post-translational modifications [38]. Moreover, the molecular modulations supporting the tumorigenic signaling at 8 weeks were confirmed by the IPA analysis of RNA sequencing data obtained from the macrodissected ACFs of Winnie-*Apc^Min/+^*-TNF-KO mice relative to the Winnie-*Apc^Min/+^* mice. Surprisingly, the absence of TNF induced a more aggressive phenotype for the tumoral lesions in Winnie-*Apc^Min/+^*-TNF-KO mice because of the altered mechanisms involved in carcinogenesis, tumor microenvironment, and cellular remodeling; thus, this observation supports the presence of the “advanced malignant tumor” disease and functions among the top regulator effect networks in the context of gastrointestinal disease, which was identified by the IPA. Specifically, the more aggressive phenotype of Winnie-*Apc^Min/+^*-TNF-KO mice could be supported, among others, by the TGFβ signaling. In fact, in line with the IPA-predicted inhibition for TGFβ as an upstream regulator, TGFβR2 greatly decreased in Winnie-*Apc^Min/+^*-TNF-KO as compared with Winnie-*Apc^Min/+^* mice. This data could be explained by considering the relationship between the TGFβ gene and the expression of TNF as reported in the literature, even if different pathological contexts had been studied [39,40,41]. Our TGFβR2 expression data in Winnie-*Apc^Min/+^*-TNF-KO was also supported by a paper describing the development of lethal inflammatory disease and invasive CRC in a mutant APC model of colon carcinogenesis, which was characterized by an epithelial truncation of TGFβR2 [42]. Thus, the loss of TGFβ signaling, specifically in colon epithelial cells, promotes a strong inflammatory response and supports tumor progression. TGFβ exerts key roles in inflammation, carcinogenesis, and the tumor microenvironment [43]. More generally, it both acts on colonic epithelial cells by inhibiting cell proliferation and promoting differentiation and apoptosis [44] and acts on the intestinal immune response by suppressing intestinal immune cells in the stroma and inducing immune tolerance against luminal bacterial antigens [45,46]. Thus, the tight interaction of the inflammatory pathways with the cellular pathways linked to cancer biology features the role of TGFβ signaling modulations in promoting an aggressive CAC phenotype in Winnie-*Apc^Min/+^*-TNF-KO mice. Of note, this interaction implies an accurate consideration for the treatment of CAC patients; in fact, the use of TGFβ inhibitors may result in a worse outcome through the enhancement of the inflammatory responses. However, a deep insight into the TGFβ signaling paradoxical dynamics related to the disease stage (early vs. late) is needed to clarify its role in CAC pathogenesis.

## 4. Materials and Methods

### 4.1. Animal Studies

Our investigations were performed under the relevant animal protocol, which was approved by the Institutional Animal Care Committee of the National Institute of Gastroenterology “S. de Bellis” (Organism engaged for compliance of Animal Wellbeing, OPBA). All of the animal experiments were carried out according to the national guidelines of the Italian Directive n. 26/2014 and were approved by the Italian Animal Ethics Committee of the Ministry of Health—General Directorate of Animal Health and Veterinary Drugs (DGSAF- Prot. 768/2015-PR 27/07/2015). All animals were maintained in a controlled environment (20–22 °C, 12 h light and 12 h dark cycles, and 45–55% relative humidity).

The new murine transgenic line Winnie-*Apc^Min/+^*-TNF-KO was created by breeding Winnie, *Apc^Min/+^* mice, and *Tnf* knockout murine line on a C57BL/6J (WT) background. The WT, *Apc^Min/+^* mice, and *Tnf* knockout murine lines were purchased from Jackson Laboratories (C57BL/6J, Stock No. 000664, C57BL/6J-ApcMin/J, Stock No. 002020, B6.129S-Tnftm1Gkl/J, Stock No: 005540, respectively) (Bar Harbor, ME, USA).The Winnie mice were obtained from the University of Tasmania (Dr. R. Eri’s laboratory). 

Mice were sacrificed at 5 or 8 weeks of age, and their colons were removed to evaluate the presence of dysplastic lesions. The colon lengths, weights, and ratio between the colon length and total body weight were measured as indicators of colonic inflammation. 

### 4.2. Histology

Tissue sections from the large intestine were fixed in 10% buffered formalin, dehydrated, and paraffin-embedded. We stained 3-micrometer thick sections from the proximal, medial, and distal colon regions using a hematoxylin-eosin standard protocol. Colonic tissue sections were evaluated for neoplasia. Periodic acid–Schiff (PAS) staining on distal colon sections was performed to identify mucins. Observations and imaging were performed with a Nikon Eclipse Ti2 microscope (Nikon, Tokyo, Japan). 

### 4.3. RNA Extraction from Colon Tissue and qPCR Analysis

The total RNA was isolated from the distal part of the large intestine of mice. The RNA was extracted using TRIzol^®^ reagent (Thermo Fisher Scientific, Waltham, MA, USA) according to the manufacturer’s instructions. The RNA quality was checked by the A260/280 ratio. The total RNA (1 µg) was reverse-transcribed using an iScript cDNA Synthesis kit (Biorad, Hercules, CA, USA) using random primers for cDNA synthesis. Starting at 10 ng of cDNA for sample, the gene expression analysis was performed testing four animals for each experimental group and using Colonic neoplasms Tier 1 M96 (Cat.#100-36551, Biorad, Hercules, CA, USA). Each plate contained internal controls to check for DNA contamination, PCR efficiency, RNA integrity, and cDNA quality; for the latter, a control RNA template was added during the reverse transcription reaction (Supplementary Figure S5). A real-time PCR was performed on a CFX96 System (Biorad, Hercules, CA, USA) using the following thermal cycler protocol: activation step of 95 °C for 2 min and 1 cycle; denaturation step of 95 °C for 5 s; annealing/extension step of 60 °C for 30 s. The last two steps were repeated for a total of 40 cycles. A melt curve was performed at the end of the real-time reaction. Gene name, gene symbols, Entrez Gene ID, amplicon sequence and length, and assay design are reported in Supplementary Table S3. The expression of all target genes was calculated relative to the *Gapdh* expression using the 2^−ΔCt^ method. 

### 4.4. RNA Extraction from Macrodissected Tumors

Tumor specimens were macrodissected by a pathologist starting from the histological analysis of the hematoxylin-eosin (HE)-stained slides. Specifically, the tumoral area was circled on HE slides and used to perform a manual macrodissection on 10-micrometer thick unstained sections that were corresponding and consecutive to HE slides. Under the guidance of a microscope, the pathologist first removed the normal tissue that externally surrounded the circled area and then scraped off the slide, i.e., the tumoral area, using a sterile fine blade collecting the dissected material into RNase free tubes. The procedure was performed taking care to not carry more than 30% of normal tissue within the sample. RNA was extracted from the macrodissected tissues using the Qiagen RNeasy FFPE kit (Hilden, Germany) according to the manufacturer’s instructions.

### 4.5. RNA Exome Sequencing

Next-generation sequencing experiments, comprising sample quality control, were performed by Genomix4life SRL (Baronissi, Salerno, Italy). Indexed libraries were prepared from 20 ng of purified RNA with the TruSeq RNA Exome sample prep kit (Illumina, San Diego, CA, USA) according to the manufacturer’s instructions. Libraries were quantified using the TapeStation 4200 system (Agilent Technologies, San Diego, CA, USA) and Invitrogen Qubit fluorometer (Thermo Fisher Scientific, Waltham, MA, USA) and were then pooled such that each index-tagged sample was present in equimolar amounts, with the final concentration of the pooled samples being 2 nM. Pooled samples were subjected to cluster generation and sequencing using an Illumina NextSeq 500 system (Illumina, San Diego, CA, USA) in a 2 × 75 paired-end format at a final concentration of 1.8 pmol.

Sequencing data are available at the ArrayExpress database under the accession number E-MTAB-12356.

### 4.6. Statistical and Bioinformatics Analysis

Fastq underwent quality control using the FastQC tool (http://www.bioinformatics.babraham.ac.uk/projects/fastqc; accessed on 19 October 2022). The mapping of paired-end reads was performed using the bioinformatics tool STAR (version 2.5.1a) [47], with the standard parameters on the reference genome assembly mm10 having been obtained from Ensembl (http://www.ensembl.org/Mus_musculus/Info/Index; accessed on 19 October 2022). The quantification of transcripts expressed for each replicate of the sequenced samples was performed using the featureCount algorithm [48]. The Bioconductor package DESeq2 [49] was used to normalize the data and was then used to perform the differential expression analysis. Hierarchical clustering and PCA was created using the packages ComplexHeatmap and ggplot2 in R software.

Networks and biological functions on differential expressed genes were assessed using IPA software (Qiagen, Hilden, Germany). The canonical pathways and networks were the results of the IPA core analysis.

For all other analyses, statistical evaluations were performed using the GraphPad Prism statistical software, release 8.4. Data were expressed as the mean ± SEM, and results were obtained from three consecutive and independent experiments. Before proceeding to statistical comparisons, the data normality and homogeneity of variance were checked by the Shapiro–Wilk test and F test, respectively. Unless specifically described, all data were analyzed and compared by paired, two-tailed Student’s *t*-tests. Results were considered statistically significant at *p* < 0.05. 

## 5. Conclusions

The data reported in this study point out a dual role for TNF in CAC, promoting the onset of neoplastic lesions at early time points and controlling their later progression; however, additional studies are needed to verify the mechanisms identified by our work. Importantly, the Winnie-*Apc^Min/+^*-TNF-KO mice could be an effective model for the discovery of new biomarkers to be used for the early detection of young UC patients unresponsive to anti-TNF therapy as well as for the prediction of patient prognosis in this subgroup of UC patients that rapidly develop CAC. Thus, our results could be useful not only in reducing the economic costs but also in helping the design of appropriate precision medicine protocols to prevent CAC onset and/or progression.

In conclusion, the depletion of TNF from the colon of CAC-developing mice started a cascade of events, which induced the onset of tumor lesions in the distal colon at intermediate time points; these lesions are characterized by a more aggressive phenotype. This peculiar mechanism is supported by TGFβ as defined by our molecular classification. In summary, this work provides crucial insights towards the correct management of anti-TNF therapy in UC patients.

## Figures and Tables

**Table 1:** Histological analysis of 5-week-old Winnie-*APC^Min/+^*-TNF-KO mice and control littermates. Incidence and multiplicity ± SEM of proximal, medial, and distal colon were calculated for non-dysplastic and dysplastic ACFs. The score for dysplastic ACFs was calculated relative to all groups, and each group of neoplastic lesions was classified according to the dimension and grading in the unicryptic lesions and microadenoma: >1≤5 crypts LG; TNF: tumor necrosis factor; ACFs: aberrant crypt foci; LG: low grade.

5 Week	Non Dysplastic ACFs		Dysplastic ACFs					
			All Groups		Unicryptic Lesion		Microadenoma >1 ≤ 5−LG	
Genotype (nr. Mice)	Incidence (%)	Multiplicity (Mean ± SEM)	Incidence (%)	Multiplicity (Mean ± SEM)	Incidence (%)	Multiplicity (Mean ± SEM)	Incidence (%)	Multiplicity (Mean ± SEM)
**PROXIMAL COLON**								
TNF-KO (8)	12.5	0.125 ± 0.125	0	0	0	0	0	0
Winnie-TNF-KO (8)	12.5	0.2 ± 0.25	0	0	0	0	0	0
*APC^Min/+^*-TNF-KO (10)	0	0	0	0	0	0	0	0
Winnie-*APC^Min/+^*-TNF-KO (10)	0	0	10	0.1 ± 0.1	0	0	100	0.1 ± 0.1
**MEDIAL COLON**								
TNF-KO (8)	12.5	0.125 ± 0.125	0	0	0	0	0	0
Winnie-TNF-KO (8)	0	0	0	0	0	0	0	0
*APC^Min/+^*-TNF-KO (10)	0	0	10	0.1 ± 0.1	100	0.1 ± 0.1	0	0
Winnie-*APC^Min/+^*-TNF-KO (10)	0	0	20	0.2 ± 0.13	50	0.1 ± 0.1	50	0.1 ± 0.1
**DISTAL COLON**								
TNF-KO (8)	12.5	0.125 ± 0.125	0	0	0	0	0	0
Winnie-TNF-KO (8)	0	0	12.5	0.11 ± 0.13	0	0	100	0.11 ± 0.13
*APC^Min/+^*-TNF-KO (10)	0	0	30	0.6 ± 0.4	50	0.3 ± 0.21	50	0.3 ± 0.21
Winnie-*APC^Min/+^*-TNF-KO (10)	0	0	90	1.2 ± 0.26	16.7	0.2 ± 0.13	83.3	1 ± 0.25

**Table 2:** Top upstream regulators from the IPA core analysis of differentially expressed genes between 8-wk-old Winnie- *APC^Min/+^*-TNF-KO mice and Winnie-*APC^Min/+^* mice.

Top Upstream Regulators		
Name	*p*-Value	Predicted Activation
MYC	1.07 × 10^−17^	
Beta-estradiol	6.23 × 10^−16^	Inhibited
KRAS	8.47 × 10^−16^	
APP	2.81 × 10^−14^	Inhibited
TGFβ	9.12 × 10^−14^	Inhibited

## Data Availability

The data presented in this study are available upon reasonable request from the corresponding authors. Sequencing data are available at the ArrayExpress database under the accession number E-MTAB-12356.

## Supplementary Materials

The following supporting information can be downloaded at: https://www.mdpi.com/1422-0067/23/23/15145/s1.
