# Supplementary material for: Deletion of TNF in Winnie-APCMin/+ Mice Reveals Its Dual Role in the Onset and Progression of Colitis-Associated Colorectal Cancer
Source: Int J Mol Sci. 2022 Dec 2;23(23):15145. doi: 10.3390/ijms232315145 (PMC9737576; doi:10.3390/ijms232315145)
Supplement: Supplementary file 1 [file ijms-23-15145-s001.zip › ijms-2050416-supplementary.pdf]

Supplementary Data Captions:

Supplementary Table S1: Histological analysis of 8-wk-old Winnie-*APC*$^{Min/+}$ and Winnie-*APC*$^{Min/+}$-TNF-KO mice and the control littermates. Incidence and multiplicity ± SEM of proximal, medial, and distal colon were calculated for non-dysplastic and dysplastic ACFs. The score for dysplastic ACFs was calculated relative to all groups and each group of neoplastic lesions was classified according to dimension and grading in unicryptic lesions; microadenoma >1 ≤5 crypts LG; microadenoma >5 crypts LG, and microadenoma >5 crypts HG. TNF: tumor necrosis factor; ACFs: aberrant crypt foci; LG: low grade; HG: high grade.

8 WEEK	Non dysplastic ACFs		Dysplastic ACFs									
			All groups		Unicryptic lesion		Microadenoma >1≤5 - LG		Microadenoma >5 - LG		Microadenoma >5 - HG	
Genotype (nr. mice)	Incidence (%)	Multiplicity (mean ± SEM)	Incidence (%)	Multiplicity (mean ± SEM)	Incidence (%)	Multiplicity (mean ± SEM)	Incidence (%)	Multiplicity (mean ± SEM)	Incidence (%)	Multiplicity (mean ± SEM)	Incidence (%)	Multiplicity (mean ± SEM)
**PROXIMAL COLON**												
TNF-KO (8)	0	0	0	0	0	0	0	0	0	0	0	0
Winnie-TNF-KO (8)	12.5	0.37 ± 0.37	0	0	0	0	0	0	0	0	0	0
APC$^{Min/+}$-TNF-KO (8)	0	0	12.5	0.13 ± 0.13	100	0.13 ± 0.13	0	0	0	0	0	0
Winnie-*APC*$^{Min/+}$-TNF-KO (5)	40	0.4 ± 0.2	0	0	0	0	0	0	0	0	0	0
**MEDIAL COLON**												
TNF-KO (8)	0	0	0	0	0	0	0	0	0	0	0	0
Winnie-TNF-KO (8)	12.5	0.25 ± 0.25	0	0	0	0	0	0	0	0	0	0
APC$^{Min/+}$-TNF-KO (8)	0	0	62.5	0.63 ± 0.18	60	0.38 ± 0.13	40	0.25 ± 0.09	0	0	0	0
Winnie-*APC*$^{Min/+}$-TNF-KO (5)	20	0.2 ± 0.2	80	1.6 ± 0.68	25	0.4 ± 0.24	75	1.2 ± 0.58	0	0	0	0
**DISTAL COLON**												
TNF-KO (8)	0	0	0	0	0	0	0	0	0	0	0	0
Winnie-TNF-KO (8)	12.5	0.13 ± 0.13	0	0	0	0	0	0	0	0	0	0
APC$^{Min/+}$-TNF-KO (8)	0	0	25	0.25 ± 0.16	50	0.13 ± 0.13	50	0.13 ± 0.13	0	0	0	0
Winnie-*APC*$^{Min/+}$-TNF-KO (5)	0	0	100	6.6 ± 1.56	3	0.2 ± 0.2	75.8	5 ± 0.95	18.2	1.2 ± 0.37	3	0.2 ± 0.2

Supplementary Table S2: Top Regulator Effect Networks from IPA core analysis of differentially expressed genes between 8-wk-old Winnie-*APC*$^{Min/+}$-TNF-KO mice and Winnie-*APC*$^{Min/+}$ mice. TNF: tumor necrosis factor; IPA: ingenuity pathway analysis.

Top Regulator Effect Networks			
**ID**	**Regulators**	**Disease and Functions**	**Consistency Score**
1	FOXC2, FOXF2, HSPA9, PAK1, TLN1	Cancer of cells, Cell movement of tumor cell lines (+6 more)	21,019
2	FOXC2, HSPA9, mir-29, NRG1, NSUN6, Pkc(s), STK40	Advanced malignant tumor, Cancer of cells (+3 more)	20,677
3	CR1L, FOXC2, FOXF2, GMNN, HSPA9, MRTFA, NRG1, NSUN6 (+6 more)	Advanced malignant tumor (+5 more)	18,543
4	Brd4, FOXC2, FOXF2, HSPA9, IPMK, ITGA11, MAFB, PAK1, PKD1 (+3 more)	Cancer of cells, Cell movement of tumor cell lines (+6 more)	18,411
5	CR1L, FOXC2, FOXF2, HSPA9, MRTFA, MRTFB, PSMB11, SUCNR1 (+1 more)	Cancer of cells, Cell movement of tumor cell lines (+3 more)	16,554

Supplementary Table 3: Gene name, gene symbols, Entrez Gene ID, amplicon sequence and length, and assay design for the mRNAs spotted on the pre-custom plates for colorectal cancer (colonic neoplasms Tier 1 M96, cat.#100-36551, Biorad, Hercules, CA, USA) used for gene expression analysis of the whole distal colon from Winnie-*APC*$^{Min/+}$ and Winnie-*APC*$^{Min/+}$-TNF-KO mice at 5 and 8 weeks. TNF: tumor necrosis factor.

Gene Name	Gene Symbol	Entrez Gene ID	Amplicon Context Sequence	Amplicon Length	Assay Design
ATP-binding cassette, sub-family B (MDR/TAP), member 1A	Abcb1a	18671	AGAGGACCTTAAGGGAAGAGCAGACAAGAACTTCTCAAAGATGGGCAAAAAGAGTAAAAAGGAGAAGAAAGAAAAGAAACCAGCAGTCAGTG	92	Intron-spanning
Apolipoprotein E	Apoe	11816	CTGTCAGCAATGTGACCAACAGCACGGCCCACAGAGCCTTCATCTTCGCAATTGTGATTGG	61	Exonic
Cyclin-dependent kinase inhibitor 1A (P21)	Cdkn1a	12575	CCTCACTCTGTGTGTCTTAATTATTATTTGTGTTTTAATTTAAACGTCTCCTGTATATACGC	62	Exonic
Cysteine rich protein 61	Cyr61	16007	CCAATTGCGATTAACTCATTGTTTCTCGTTAACTCCACCTCCGAGGCATCGAGTCCGAGGAGGTCATCCTGGTCGTCCAGGGAGTCCTTAATGCTGTCTTCATCA	105	Exonic
Growth arrest and DNA-damage-inducible 45 beta	Gadd45b	17873	ATATCGCTCTGCAGATTCACTTCACCCTGATCCAGTCGTTCTGCTGCGACAATGACATTGACATCGTCCGGGTATCAGGCATGCAGAG	88	Exonic
Interleukin 1 beta	Il1b	16176	TTGTCGTTGCTTGGTTCTCCTTGTACAAAGCTCATGGAGAATATCACTGGAGAAAGGAGAGGAGTCTTGTTGGTTGATATTCTGTCCATTGAGGT	95	Intron-spanning
Antigen identified by monoclonal antibody Ki 67	Mki67	17345	TCTTGGTGCATACAATGTCTTCATCCTCCTTCAGAGTTTTTGCATCTTTCTTGGTTCCACGAGTTACCCTTGGCTTAGGTTCACTGTCCAA	91	Intron-spanning
Phosphatidylinositol 3-kinase, catalytic, alpha polypeptide	Pik3ca	18706	TGGAGAAGCTCATCAACCTAACGGACATCCTTAAGCAGGAGAAGAAGGATGAGACACAAAAGGTACAGATGAAGTTTTTGGTTGAACAGATGAGACAG	98	Intron-spanning
Ribonucleotide reductase M2	Rrm2	20135	AGCGATTTAGCCAAGAAGTTCAAGTTACAGAGGCCCGCTGTTTCTATGGCTTCCAAATTGCCATGGAAAACATACACTCTGAAATGTACAGTCTCCTTATTGACACTTACATTAAAGATCCCAAGGAAAGAGAATATCTCTTCAATGCTATTGAAACAATGCCTTGTGTG	170	Intron-spanning
Secreted phosphoprotein 1	Spp1	20750	TTCTGTGGACTCGGATGAATCTGACGAATCTCACCATTCGGATGAGTCTGATGAGACCGTCACTGCTAGTACACAAGCAGACACTTTCACTCCAATCGTCCCTACAGTCGAT	112	exonic
Thymidylate synthase	Tyms	22171	TCAGCGGCTCTATATGATTCAGGTAAATATGTGCATCTCCCAAAGTGTGGACAAAATCACCTGGCTAAAAATGTCTGCAGGCCTGTGATATGTGCAATCATGTAGGTGAGCAGAGCATA	119	Intron-spanning
TATA box binding protein	Tbp	21374	GGAGAATCATGGACCAGAACAACAGCCTTCCACCTTATGCTCAGGGCTTGGCCTCCCCACAGGGCGCCATGACTCCTGGAATTCCCATCTTTAGTCCAATGA	102	Intron-spanning
Actin, alpha 2, smooth muscle, aorta	Acta2	11475	CATAGATAGGCACGTTGTGAGTCACACCATCTCCAGAGTCCAGCACAATACCAGTTGTACGTCCAGAGGCATAGAGGGACAGCACAGCCTGAATAGCCACATACATGGCGGGGACATTGAAGGTCTCAAACATAATCTGGGTCATTTTCTCCCGGTTGGCCTTAGGGTTCAGTGGTGC	178	Intron-spanning
Caveolin 1, caveolae protein	Cav1	12389	GTGACTGAGAAGCAAGTGTATGACGCGCACACCAAGGAGATTGACCTGGTCAACCGCGACCCCAAGCATCTCAACGACGACGTGGTCAAGATTGACTTTGAAGATGTGATTGCAGAACCA	120	Intron-spanning
Cyclin-dependent kinase inhibitor 2A	Cdkn2a	12578	TATCGCACGATGTCTTGATGTCCCCGCTCTTGGGCCAAGTCGAGCGGCAGGCGACCCCAGGCATCGCGCACATCCAGCCGAGCCCCTGACCCGTGCAGCACCACCAGCGTGTCCAGGAAGCCTTCCCGCGCTGCGTCGTGCACCGGGCGGGAGAAGGTAGTGGGGTCCTCGCAGTTCGAATCTGCACCGTAGTTG	195	Exonic
Epidermal growth factor receptor	Egfr	13649	AACACTCTGGTCTGGAAGTATGCAGATGCCAATAATGTCTGCCACCTATGCCACGCCAACTGTACCTATGGATGTGCTGGGCCAGGTCTTCAAGGATGTGAAGTGTGG	108	Intron-spanning
Glutathione S-transferase, mu 1	Gstm1	14862	AGGAACTCAGAGTAGAGCTTCATTTTCTCAGGGATGGTCTTCAAGAACTCTGGCTTCTGCTTCTCAAAGTCAGGGTTGTAACAGAGCATGATGAGCTG	98	Intron-spanning
Integrin beta 1 (fibronectin receptor beta)	Itgb1	16412	GCTAATCATCGATGCCTACAACTCTCTTTCTTCAGAAGTCATTCTGGAAAATAGCAAATTGCCAGACGGAGTAAC	75	Intron-spanning
Myelocytomatosis oncogene	Myc	17869	CTAGTGCTGCATGAGGAGACACCGCCCACCACCAGCAGCGACTCTGAAGAAGAGCAAGAAGATGAGGAAGAAATTGATGTGGTGTCTGT	89	Intron-spanning
Plasminogen activator, urokinase receptor	Plaur	18793	TCCATAGCAACCAGACCTTTCACTTCCTGAAGTGTTGCAACTACACCCACTGCAATGGTGGCCCAGTTCTGGATCTTCAGAGCTTTCCACCGAATGGCTTCCAGTGTTACAG	112	Intron-spanning
S100 calcium binding protein A4	S100a4	20198	ATTCAGCACTTCCTCTCTCTTGGTCTGGGTCTCAACGGTTACCATGGCAAGACCCTTGGAGGAGGCCCTGGATGTAATTGTGTCCACCTTCC	92	Intron-spanning
Telomerase reverse transcriptase	Tert	21752	AGGCTCTTCTTCTACCGTAAGAGTGTGTGGAGCAAGCTGCAGAGCATTGGAGTCAGGCAACACCTTGAGAGAGTGCGGCTACGGGAGCTGTCACAAGAGGAGGTCAGG	108	Intron-spanning
Ubiquitin B	Ubb	22187	TGGCATTACTCTGCACTCTAGCCACTTGCCCCAATTTAAGTTTAGAAATTACAAGTTTCAATAATAGCTGAACCTCTGT	79	Exonic
Glyceraldehyde-3-phosphate dehydrogenase	Gapdh	14433	AACCTGGTCCTCAGTGTAGCCCAAGATGCCCTTCAGTGGGCCCTCAGATGCCTGCTTCACCACCTTCTTGATGTC	75	Exonic
Actin, beta	Actb	11461	CAACATAGCACAGCTTCTCTTTGATGTCACGCACGATTTCCCTCTCAGCTGTGGTGGTGAAGCTGTAGCCACGCTCGGTCAGGATCTTCATGAGGTAGTCTGTCAGGTC	109	Exonic
Cyclin A2	Ccna2	12428	GTTCACTGGCTTGTCTTCTAAGACAATAGACATATCCATAGCATGTGGTGATTCAAAACTGCCATCCATTGGATAGTCAAGAGGTGTCA	89	Intron-spanning
CCAAT/enhancer binding protein (C/EBP), beta	Cebpb	12608	GGCCCTGAGTAATCACTTAAAGATGTTCCTGCGGGGTTGTTGATGTTTTTGGTTTTGTTTTTGTTTTTTGTTTTGTTTTGTTTTTTTTTTTGGTCTTATTATTTTTTTGTATTATATAAAAAAGTTCTATTTCTATGAGAAAAGAGGCGTATGTATATTTTGAGAACCTTTTCCGTTTCGAGCATTAAAGTGAA	195	Exonic
Early growth response 1	Egr1	13653	GACTCTGCTGTGACATTAGGTTTGAAGCATTTTTTTTTTCAAGCAGCAGTCCTAGGTATTAACTGGAGCATGTGTCAGAGTGTTGTTCCGTTAA	94	exonic
Hypoxia inducible factor 1, alpha subunit	Hif1a	15251	GGACGATGAACATCAAGTCAGCAACGTGGAAGGTGCTTCACTGCACGGGCCATATTCATGTCTATGATACCAACAGTAACCAACCTCAGT	90	Intron-spanning
Jagged 1	Jag1	16449	ACAGGTCCCGCTATTGTAACAAGGATGGGGACTGCAGTCGTTGGTATTTTGAGTACAAATAGGCCCCTCCCAGCCTTCTTTGCAGACACAGGTGAAGGAGTCTCCATTGACCACGCAGGTACCTCCATTATGACAGGGGTTCGGCAGGCAGCTACTGTTTCTAGCTATATTACAGGTTGTTCCTTCCC	188	Intron-spanning
NME/NM23 nucleoside diphosphate kinase 1	Nme1	18102	CTCTGCGCTCTTTACAGAATCGCTGCCATGAATGATGTTCCTGCCAACTTGGATGCAGAAGTCTCCTCGTATGGTCCCAGGCTTAGAGTCTGCGGGGTTGGTCTCTCCAAGCATCACGCGGCCTGTCTTCACCACATTCAGAC	143	Intron-spanning
Polo-like kinase 2 (Drosophila)	Plk2	20620	AGTGTTACTGACATTCGAAGACCTCGGCTCTACCTCCTGCAGTGGTTAAAGTCTGATAAAGCCTTAATGATGCTCTTCAATGACGG	86	Exonic
Serine (or cysteine) peptidase inhibitor, clade E, member 1	Serpine1	18787	CGAACCACAAAGAGAAAGGATCGGTCTATAACCATCTCCGTGGGGGCCATGCGGGCTGAGATGACAAAGGCTGTGGAGGAAGACGCCACTGTGCCGCTCTCGTTTACCTCGATCCTGACCTT	122	Intron-spanning
Transforming growth factor, beta 1	Tgfb1	21803	AATCAAGTGTGGAGCAACATGTGGAACTCTACCAGAAATATAGCAACAATTCCTGGCGTTACCTT	65	Intron-spanning
Ubiquitin C	Ubc	22190	GGATCTTTGCCTTGACATTCTCAATGGTGTCACTGGGCTCGACCTCCAGGGTGATGGTCTTACCAGTTAAGGTTTTCACAAAGATCTGCATCGTCTCTCTCACGGAGTTGTTTCAC	116	Intron-spanning
Hypoxanthine guanine phosphoribosyl transferase	Hprt	15452	TTATCAGACTGAAGAGCTACTGTAATGATCAGTCAACGGGGGACATAAAAGTTATTGGTGGAGATGATCT	70	Intron-spanning
Thymoma viral proto-oncogene 1	Akt1	11651	AATACCTGGTGTCAGTCTCAGAGGTGACCTGGGGCTTGAAAGGTGGGCTCAGCTTCTTCTCATACACATCCTGCC	75	Intron-spanning
Cyclin D1	Ccnd1	12443	CAGAAGGGCTTCAATCTGTTCCTGGCAGGCACGGAGGCAGTCCGGGTCACACTTGATGACTCTGGAAAGAAAGTGC	76	Intron-spanning
CUGBP, Elav-like family member 2	Celf2	14007	CATTCAGAGCCGCCATACTGTTTATGGTACCTGCTAGTGCATTAATATTATTCAATCCGACAGTGGCTCCAGCCAATCCTTGTAGAGTCCCAAGAGAGGTCAAGGAATTCA	111	Intron-spanning
Enolase 1, alpha non-neuron	Eno1	13806	GCGTCCACTGGCATCTACGAGGCCCTAGAACTCCGAGACAATGATAAGACCCGCTTCATG	60	exonic
Heat shock protein 1A	Hspa1a	193740	AATTGACCCGAGTTCAGGATGGTTGTGTAAAAACTGAACACATGCTGGTGCTGTCACTTCACCTCCAAGTTCACCAACCTGGAAACAAGTCCTA	94	exonic
V-Ki-ras2 Kirsten rat sarcoma viral oncogene homolog	Kras	16653	ATAGAAGGCATCGTCAACACCCTGTCTTGTCTTTGCTGAGGTCTCAATGAACGGAATCCCGTAACTCCTTGCTAACTCCTGA	82	Intron-spanning
Notch gene homolog 1 (Drosophila)	Notch1	18128	AGTCTTCAGACTCCTTGCATACCCCGCTGTTTTTGCAGGGGCTGGTAGCACATGGGGCCAACACCACCTCACACGTGGCTCCTGTATATGGCAGAGGACAGT	102	Intron-spanning
Peroxisome proliferator activated receptor alpha	Ppara	19013	ACGAAGCCTACCTGAAGAACTTCAACATGAACAAGGTCAAGGCCCGGGTCATACTCGCGGGAAAGACCAGCAACAACCCGCCTTTTGTCATACATGACATGGAGACCTTGTGTA	114	Intron-spanning
Secreted frizzled-related protein 1	Sfrp1	20377	AATACCACGGAAGCCTCTAAGCCCCAAGGTACAACCGTGTGTCCTCCATGCGACAACGAGTTGAAGTCAGA	71	Intron-spanning
Tissue inhibitor of metalloproteinase 1	Timp1	21857	GGCATCCTCTTGTTGCTATCACTGATAGCTTCCAGTAAGGCCTGTAGCTGTGCCCCACCCCACCCACAGACAGCCTTCTGCAACTCGGACCTGGTCATAAGGGCTAAATTCATGGGTTCCCCAGAAATCAACGAGACCACCTTATACCA	149	Intron-spanning
Vitamin D receptor	Vdr	22337	GTCATAGCGTTGAAGTGGAAGCCCGTGGCTCGGTCTCCACACACTCCACAGATCCGAGGCACATTCCGGTCAAAGTCACCAGGGTCAGGCAGGGAGGTGCTGGCTGCCATTGCCTCCATCCCTGAAGGAGCAGGGGCGAGCAGAGCCCAAGGGTGCTCTCCTGGGAAGACTCACAGATCTG	181	Intron-spanning
Thymoma viral proto-oncogene 3	Akt3	23797	GCAACCTTCCACACATCATTTCATACATGACAACACCTAAGCCCCACCAGTCCACGGCTCGGCCATAGTCATTATCTTCTAATACCTCTGGTGCCAGGTACTCTGGTGTGCCACAG	116	Intron-spanning
Cyclin D2	Ccnd2	12444	GGGTACATGGCAAACTTGAAGTCGGTAGCGCACAGAGCGATGAAGGTCTGCGCATGCTTGCGGATCAGGGACAGCTTCTCCTTTTGCTGGGGCAGCTTGCGAAGGATGTGCTCAATGAAGTC	122	Intron-spanning
Collagen, type I, alpha 1	Col1a1	12842	GCATGGCCAAGAAGACATCCCTGAAGTCAGCTGCATACACAATGGCCTAAGGGTCCCCAATGGTGAGACGTGGAAACCCGAGGTATGCTTGATCTGTATCTGCC	104	Intron-spanning
V-erb-b2 erythroblastic leukemia viral oncogene homolog 2, neuro/glioblastoma derived oncogene homolog (avian)	Erbb2	13866	ACCTCTCCTACATGCCTATCTGGAAGTACCCGGATGAGGAGGGCATATGTCAGCCATGCCCCATCAACTGCACCCACTCCCCAGTGACATTCATCATTGCAACTGTGGT	109	Intron-spanning
Interferon gamma inducible protein 30	Ifi30	65972	GACTCATAATACAGGCTGACTCTGACAGGTGGTGAAGGGGGCAATGGGCGTGGCCCCAAGAGGCACACATCGTGGGCCTTGCAGGTGGTTGTGCCTTCGGAGGATGCTTGCGAAAGGGACGCAGTGGCTGCTCTGGGAACCTCGAGTGGGAACAGCAGAAGCAGAAGGGACAAAAATGGTAGGATCGGAGACCAGGA	197	Intron-spanning
Keratin 19	Krt19	16669	GCGAGCATTGTCAATCTGTAGGACAATCTTGGAGTTGTCAATGGTGGCACCAAGAATCTTGTCGCGCAAGTCCTCGATGGTCTTAAAGTAGTGGTTGTAATCTCGG	106	Intron-spanning
Nuclear receptor subfamily 3, group C, member 1	Nr3c1	14815	AGAACTCACATCTGGTCTCATTCCAGGGCTTGAATATCCATTAGAAAACACTGAGCGGCCTGCGAAGTTCATAGCCCCCAAGGAAGTCAGGTTGTCCT	98	Intron-spanning
Peroxisome proliferator activated receptor gamma	Pparg	19016	CCTGAAGCTCCAAGAATACCAAAGTGCGATCAAAGTAGAACCTGCATCTCCACCTTATTATTCTGAAAAGACCCAGCTCTACAACAGGCCTCATGA	96	Intron-spanning
Snail homolog 1 (Drosophila)	Snai1	20613	AACTGCAAATATTGTAACAAGGAGTACCTCAGCCTGGGCGCTCTGAAGATGCACATCCGAAGCCACACGCTGCCTTGTGTCTGCACGACCTGTGGAAAGGCCTTCTCTAGGCCCTGGCTGCTTCAGGGCCACGTCCGCACCCACACTGGTGAGAAGCCATTCTCCTGCTCCCACT	175	Intron-spanning
Tissue inhibitor of metalloproteinase 2	Timp2	21858	TAATGTGCATCTTGCCATCTCCTTCTGCCTTTCCTGCAATTAGATACTCCTTCTTTCCTCCAACGTCCAGCGAGACC	77	Intron-spanning
Vascular endothelial growth factor A	Vegfa	22339	GTGCTGTAGGAAGCTCATCTCTCCTATGTGCTGGCTTTGGTGAGGTTTGATCCGCATGAT	60	exonic
Annexin A1	Anxa1	16952	CCAGATGTATCTGAAGTGATGTCTTTGGCCAGATCTCTTTTCAGCTCTTCTCTGTAGACTCTATTAATCTCTCTGATTTGTTCGTTAGATCTTGTTGTCA	100	Intron-spanning
CD44 antigen	Cd44	12505	ACCATCACGGTTGACAATAGTTATGGTAACCGGTCCATCGAAGGAATTGGGTAGGTCTGTGACTGATGTACAGTCTTCTTCAGGAGGGGCTGAGGCATTGAAGCAATATGTGTCA	115	Intron-spanning
C-reactive protein, pentraxin-related	Crp	12944	ACTGTCCTCTTAGTCCAGATCCCAGCAGCATCCATAGCCATGGAGAAGCTACTCTGGTGCCTTCTGAT	68	Exonic
Excision repair cross-complementing rodent repair deficiency, complementation group 1	Ercc1	13870	ATCCAGACTACATCCATGAACGGCTGCAGAGCCTGGGGAAGAACTTCGCCCTTCGTGTGCTGCTGGTTCAAGTGGATGTGTGCAGAGGAAGCAGGGCGGTACCTGGAGACCTACAAGG	118	Intron-spanning
Interferon gamma	Ifng	15978	GGATGCATTCATGAGTATTGCCAAGTTTGAGGTCAACAACCCACAGGTCCAGCGCCAAGCATTCAATGAGCTCATCCGAGTGGTCCACCAGCTGTTGCCGGAATCCAGCCTCAGGAAGCGGAAAAGGAGTCGCTGCTGATTCGGGGTGGGGAAGAGATTGTCCCAATAAGAATAA	175	Intron-spanning
Lectin, galactose binding, soluble 3	Lgals3	16854	AACACGAAGCAGGACAATAACTGGGGAAAGGAAGAAAGACAGTCAGCCTTCCCCTTTGAGAGTGGCAAACCATTCAAAATACAAGTCCTGGTTGAAGC	98	Intron-spanning
Peptidylglycine alpha-amidating monooxygenase	Pam	18484	ACACACCGGAACAGTCTTTGTGATTATCTCGAAAAGCACTGATGTCCCCATAGTGAACTTGAAGGACGAAGTATTTGCTTCCAGTTTCCCCTCCAACTCTGAATCCAACAC	111	Intron-spanning
Prominin 1	Prom1	19126	CCTTGGAATCAACTGAGATGTCAAAGTTCTTGTTCTGTATGAGTTTTTTGATCAAATCTAGAGGGAAGTCATTCGGCTGGACCACGTTGAGGAAGATGTGCACC	104	Intron-spanning
Superoxide dismutase 2, mitochondrial	Sod2	20656	CTCTAATCAGGACCCATTGCAAGGAACAACAGGCCTTATTCCGCTGCTGGGGATTGACGTGTGGGAGCACGCTTACTACCTTCAGTATAAAAACGTCAGACCTGACTATCTGAAAGCTATTTGGAATGTAATCAACTGGGAGAATGTTACTGAAAGATACACAGCTTGCAAGA	173	Intron-spanning
Tissue inhibitor of metalloproteinase 3	Timp3	21859	TACACAGGACTGTGCAACTTTGTGGAGAGGTGGGACCACCTCACACTGTCCCAGCGCAAGGGCCTCAATTACCGCTACCACCTGGGTTGCAATTGCAAGATCAAGTCCTGCTACTACTTG	120	Intron-spanning
Vimentin	Vim	22352	CTAACCTGGAGTCACTTCCTCTGGTTGACACCCACTCAAAAAGAACACTCCTGATTAAGACGGTTGAGACCAGAGATGGACAGGTGATCAATGAGACTTCTCAGCATCACGATG	114	Intron-spanning
Annexin A2	Anxa2	12306	GGCTGTACGACTCCATGAAGGGCAAGGGGACTCGAGACAAGGTCCTGATTAGAATCATGGTCTCTCGC	68	Intron-spanning
Cell division cycle 20 homolog (S. cerevisiae)	Cdc20	107995	CTCTCCCGAACACAAACCCCCAGCTCCTCGCAGCGCGTCAGTGCGCCTGCCGAACGCGTTCTTTTCCAGACAAGCTAAGGACACGC	86	Exonic
Connective tissue growth factor	Ctgf	14219	GGTAGGACACGAAGCTTATCTGTGATTCAAAACAAAGGAGATACTGCAGTGGGAATTGTGACCTGAGTGACTCT	74	Exonic
FMS-like tyrosine kinase 1	Flt1	14254	GATGATGAGCTGTCTTCCTTCCGTCATGTGCACAAGTTTGGGTATGTCAGTGTGCATCTCTATGAAAGGACTCCCTGCATCACTAACAAATATGTAGATTGAAGATTCCGCTT	113	Intron-spanning
Insulin-like growth factor I receptor	Igf1r	16001	TCCAACGAGCAAGTTCTTCGTTTCGTCATGGAGGGTGGCCTTCTGGACAAGCCGGACAACTGCCCTGATATGCTGTTTGAACTTATGCGCATGTGC	96	Intron-spanning
MAD2 mitotic arrest deficient-like 1 (yeast)	Mad2l1	56150	GGACTCACCTTGCTTACAACTACTGACCCCGAGCTCATAAAGTATCTCAATAATGTGGTGGAACAGCTAAAAGAGTGGCTGTACAAGTGCTCAGTTCAGAAACTGGTGGTGGTCATCT	118	Intron-spanning
Poly (ADP-ribose) polymerase family, member 1	Parp1	11545	CCGGCCATCAAGAATGAAGGAAAGAGAAAAGGTGACGAGGTGGATGGAACAGATGAAGTGGCCAAAAAGAAATCTAAGAAAGGGAAGGACAAGGATAGTAGTAAGCTGGAGAAGGC	116	Intron-spanning
Phosphatase and tensin homolog	Pten	19211	CAACCGATACTTCTCTCCAAATTTTAAGGTGAAACTATACTTTACAAAAACAGTAGAGGAGCCATCAAATCCAGAGGCTAGCAGTTCAACTTCTGTGACTCCAGA	105	Intron-spanning
Trans-acting transcription factor 1	Sp1	20683	CCAATGAGAACAGCAACAACTCCCAGGGTCCGAGTCAGTCAGGGGGCACAGGTGAACTTGACCTCACAGCC	71	Exonic
Tenascin C	Tnc	21923	CAGTCTCCTCTGTTACTTCTGTCACAATAAGGTCTTTGGGAGGGGACACCTCAGAGCAGTCTATCCCTGTGTAACCTTCATTGCAGA	87	Intron-spanning
Tyrosine 3-monooxygenase/tryptophan 5-monooxygenase activation protein, zeta polypeptide	Ywhaz	22631	GCCATGTCATCATATCGCTCTGCCTGCTCGGCCAGCTTGGCCTTCTGCACCAGCTCATTT	60	Exonic
Adenomatosis polyposis coli	Apc	11789	ATCTTCGACAAGAGCTAGAAGATAATTCCAATCATCTTACAAAACTGGAAACTGAGGCATCTAATATGAAGGAAGTACTTAAGCAGCTACAGGGAAGTATTGAAGATGAGACTATGACT	119	Intron-spanning
Cyclin-dependent kinase 1	Cdk1	12534	CAGCAACACTTCTGGAGATCGGTACCACAGCGTCACTACCTCGTGTGTGTACACTCGTATCGGTATTCCA	70	Intron-spanning
Chemokine (C-X-C motif) ligand 10	Cxcl10	15945	TTCTACTTTGTACAGTCGTTCATTAAATAGTATTTACACATACATTTTCAGGTTCCTCTGAGTATCTTGATAACC	75	Exonic
Fibronectin 1	Fn1	14268	CAGAGTCGCACTGGTAGAAGTTCCAGGAACTTGGAACTGTAAGGGCTCTTCGTCGGTGCCAACTGGTTGGCATGAAATGATGTACTCAGAACTCTCCTGGAACG	104	Intron-spanning
Insulin-like growth factor binding protein 3	Igfbp3	16009	TCCTCGGACTCACTGATGTTTCCTGGAGCAGGTTGGGAGGGGAGGTAGGTGCTTAGGCTG	60	Intron-spanning
Met proto-oncogene	Met	17295	AGTCCAACTACAGAGATGGTTTCAAATGAGTCTGTAGACTACAGAGCTACTTTTCCAGAAGACCAGTTTCCCAACTCCTCTCAGAATGGAGCATGCAGACAAG	103	Intron-spanning
Platelet derived growth factor receptor, alpha polypeptide	Pdgfra	18595	CAGACGAGAGCACAAGAAGTTATGTGATTTTGTCTTTTGAAAATAACGGCGACTACATGGACATGAAGCAAGCTGATACCACACAGTATGTCC	93	Intron-spanning
Prostaglandin-endoperoxide synthase 2	Ptgs2	19225	AATCCTTGCTGTTCCAATCCATGTCAAAACCGTGGGGAATGTATGAGCACAGGATTTGACCAGTATAAGTGTGACTG	77	Exonic
Secreted acidic cysteine rich glycoprotein	Sparc	20692	CAAAGAAGTGGCAGGAAGAGTCGAAGGTCTTGTTGTCATTGCTGCATACCTTCTCAAACTCGCCAATGG	69	Intron-spanning
Tumor necrosis factor receptor superfamily, member 1b	Tnfrsf1b	21938	GTATACATGCTTGCCTCACAGTCCGCACACACGGTGTCCGAGGTCTTGTTGCAGAAATGTTTCACATATTGGCCAGGAGGACACTTAGCACAGCACATCTGAGCCTTCCTGTCATA	116	Intron-spanning
ZW10 interactor	Zwint	52696	AGCCAAATCCAAATCTCCTTAAAGGGACAAAGAAGAACACTGGTTTGGCCTGGAACACCTTTTCTACTGAGAATGAGAAGTGGCCTGATATACTTATAGCTTGACAAGTGAGATGGTTTGATGATGTCTTCAAGAGCTAAGGAGAAGGGACTGAAAGA	158	Intron-spanning

Supplementary Figure S1: Hematoxylin and eosin staining on 3μm colon sections from proximal (A), medial (B), and distal (C) tract of 5-wk-old Winnie-*APC*$^{Min/+}$-TNF-KO mice. Images were captured at 10X (left) and 20X magnifications (right). TNF: tumor necrosis factor.

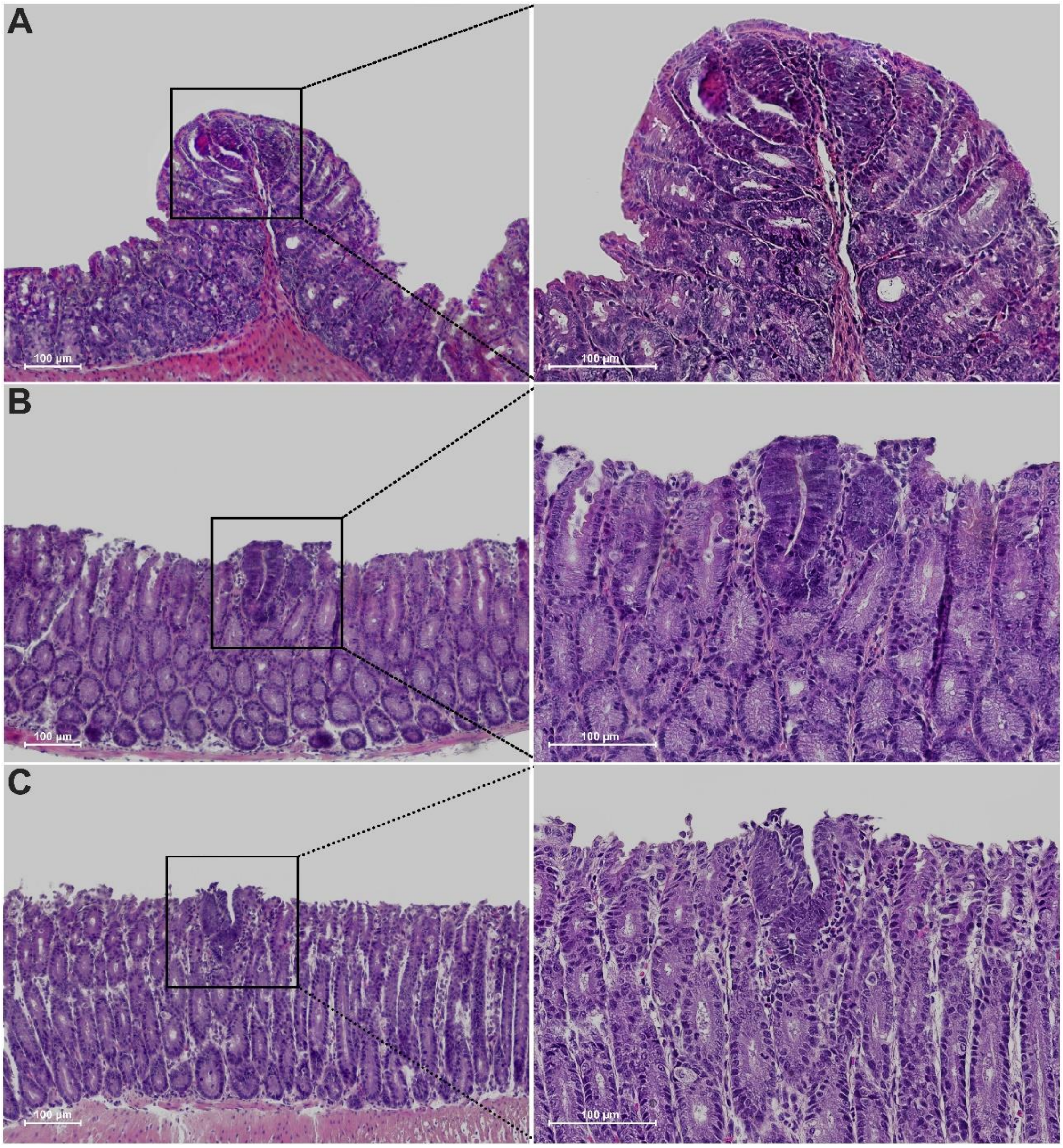

Supplementary Figure S2: PAS staining on 3μm sections from the distal colon of 5-wk-old TNF-KO (A), Winnie-TNF-KO (B), *APC*$^{Min/+}$-TNF-KO (C), and Winnie-*APC*$^{Min/+}$-TNF-KO (D). Images were captured at 20X magnifications. PAS: periodic acid–Schiff; TNF: tumor necrosis factor.

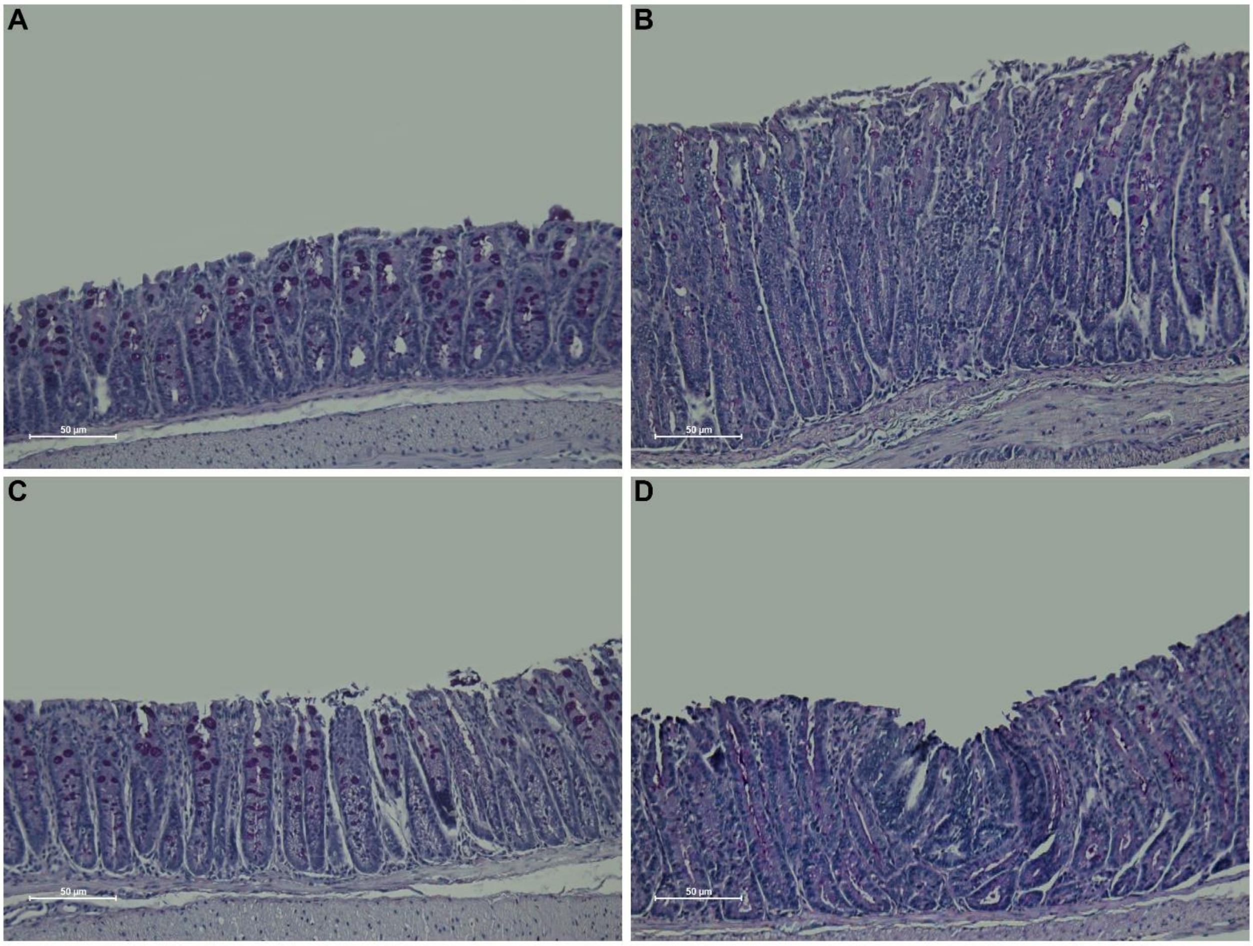

Supplementary Figure S3: Bar graphs of the most significant canonical pathways generated by IPA on differentially expressed genes in Winnie-*APC*$^{Min/+}$-TNF-KO mice relative to Winnie-*APC*$^{Min/+}$ mice at 5 weeks. IPA: ingenuity pathway analysis; TNF: tumor necrosis factor.

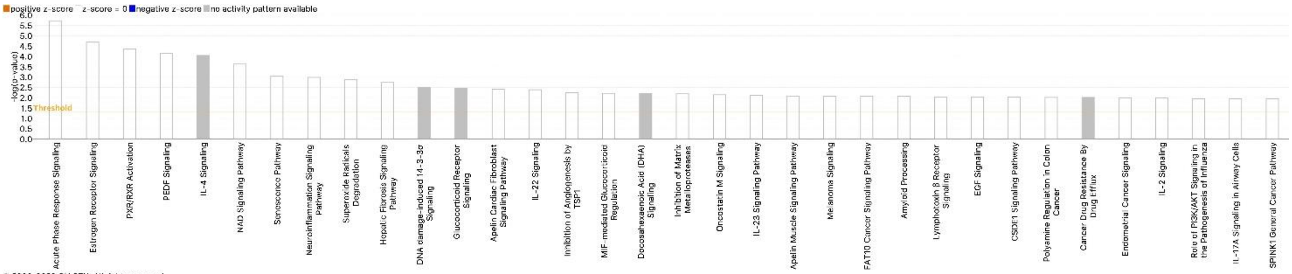

© 2000-2022 QIAGEN. All rights reserved.

Supplementary Figure S4: Bar graphs of the most significant canonical pathways generated by IPA on differentially expressed genes in Winnie-*APC*$^{Min/+}$-TNF-KO mice relative to Winnie-*APC*$^{Min/+}$ mice at 8 weeks. IPA: ingenuity pathway analysis; TNF: tumor necrosis factor.

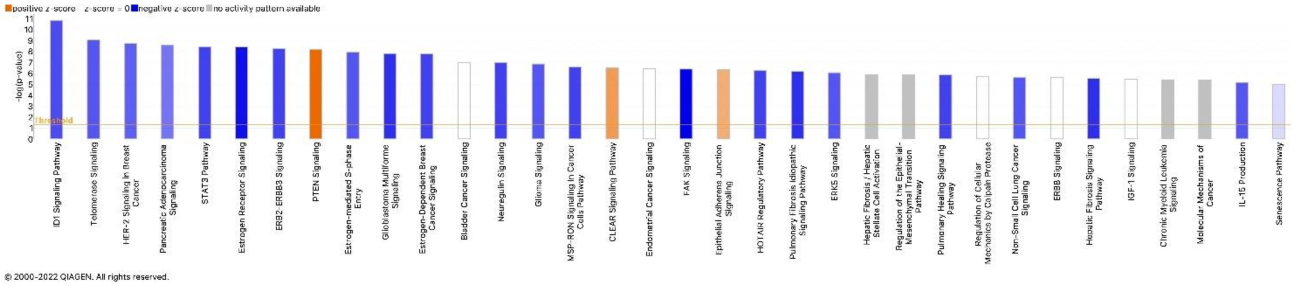

Supplementary Figure S5: Plate design of pre-custom plates for colorectal cancer (colonic neoplasms Tier 1 M96, cat.#100-36551, Biorad, Hercules, CA, USA) used for the gene expression analysis of the whole distal colon from Winnie-$APC^{Min/+}$ and Winnie-$APC^{Min/+}$-TNF-KO mice at 5 and 8 weeks.

	1	2	3	4	5	6	7	8	9	10	11	12
A	Abcb1a	Apoe	Cdkn1a	Cyr61	Gadd45b	Il1b	Mki67	Pik3ca	Rrm2	Spp1	Tyms	Tbp
B	Acta2	Cav1	Cdkn2a	Egfr	Gstm1	Itgb1	Myc	Plaur	S100a4	Tert	Ubb	Gapdh
C	Actb	Ccna2	Cebpb	Egr1	Hif1a	Jag1	Nme1	Plk2	Serpine1	Tgfb1	Ubc	Hprt
D	Akt1	Ccnd1	Celf2	Eno1	Hspa1a	Kras	Notch1	Ppara	Sfrp1	Timp1	Vdr	gDNA
E	Akt3	Ccnd2	Col1a1	Erbb2	Ifi30	Krt19	Nr3c1	Pparg	Snai1	Timp2	Vegfa	PCR
F	Anxa1	Cd44	Crp	Ercc1	Ifng	Lgals3	Pam	Prom1	Sod2	Timp3	Vim	RQ1
G	Anxa2	Cdc20	Ctgf	Flt1	Igf1r	Mad2l1	Parp1	Pten	Sp1	Tnc	Ywhaz	RQ2
H	Apc	Cdk1	Cxcl10	Fn1	Igfbp3	Met	Pdgfra	Ptgs2	Sparc	Tnfrsf1b	Zwint	RT
